# ProLoc-GO: Utilizing informative Gene Ontology terms for sequence-based prediction of protein subcellular localization

**DOI:** 10.1186/1471-2105-9-80

**Published:** 2008-02-01

**Authors:** Wen-Lin Huang, Chun-Wei Tung, Shih-Wen Ho, Shiow-Fen Hwang, Shinn-Ying Ho

**Affiliations:** 1 Institute of Information Engineering and Computer Science, Feng Chia University, Taichung, Taiwan; 2 Institute of Bioinformatics, National Chiao Tung University, Hsinchu, Taiwan; 3 Department of Management Information System, Chin Min Institute of Technology, Miaoli, Taiwan; 4 Department of Biological Science and Technology, National Chiao Tung University, Hsinchu, Taiwan

## Abstract

**Background:**

Gene Ontology (GO) annotation, which describes the function of genes and gene products across species, has recently been used to predict protein subcellular and subnuclear localization. Existing GO-based prediction methods for protein subcellular localization use the known accession numbers of query proteins to obtain their annotated GO terms. An accurate prediction method for predicting subcellular localization of novel proteins without known accession numbers, using only the input sequence, is worth developing.

**Results:**

This study proposes an efficient sequence-based method (named ProLoc-GO) by mining informative GO terms for predicting protein subcellular localization. For each protein, BLAST is used to obtain a homology with a known accession number to the protein for retrieving the GO annotation. A large number *n *of all annotated GO terms that have ever appeared are then obtained from a large set of training proteins. A novel genetic algorithm based method (named GOmining) combined with a classifier of support vector machine (SVM) is proposed to simultaneously identify a small number *m *out of the *n *GO terms as input features to SVM, where *m *<<*n*. The *m *informative GO terms contain the essential GO terms annotating subcellular compartments such as GO:0005634 (Nucleus), GO:0005737 (Cytoplasm) and GO:0005856 (Cytoskeleton). Two existing data sets SCL12 (human protein with 12 locations) and SCL16 (Eukaryotic proteins with 16 locations) with <25% sequence identity are used to evaluate ProLoc-GO which has been implemented by using a single SVM classifier with the *m *= 44 and *m *= 60 informative GO terms, respectively. ProLoc-GO using input sequences yields test accuracies of 88.1% and 83.3% for SCL12 and SCL16, respectively, which are significantly better than the SVM-based methods, which achieve < 35% test accuracies using amino acid composition (AAC) with acid pairs and AAC with dipedtide composition. For comparison, ProLoc-GO using known accession numbers of query proteins yields test accuracies of 90.6% and 85.7%, which is also better than Hum-PLoc (85.0%) and Euk-OET-PLoc (83.7%) using ensemble classifiers with hybridization of GO terms and amphiphilic pseudo amino acid composition for SCL12 and SCL16, respectively.

**Conclusion:**

The growth of Gene Ontology in size and popularity has increased the effectiveness of GO-based features. GOmining can serve as a tool for selecting informative GO terms in solving sequence-based prediction problems. The prediction system using ProLoc-GO with input sequences of query proteins for protein subcellular localization has been implemented (see Availability).

## Background

Gene Ontology (GO) [1] annotation, which describes the function of genes and gene products across species, has recently been utilized to predict protein subcellular and subnuclear localization. The prediction of protein localization is important for elucidating protein functions involved in various cellular processes. Additionally, the accomplishment of the various genome sequencing projects causes the accumulation of massive amount of gene sequence information. For example, the percentage of large-scale eukaryotic proteins with subcellular locations annotated in the Swiss-Prot database increased rapidly from 52.4% (version 49.5, released on April 18, 2006) [2] to 69.4% (version 50.7, released Sep. 11, 2006) [3]. Meanwhile, the percentage of proteins with subcellular locations annotated in the GO database increased from 44.9% [2] to 65.5% [3]. The growth of the GO database in size and popularity increases the effectiveness of GO-based features.

Some existing computation methods in literature for predicting protein localization are described below according to the used classifiers and features.

a) Mining informative features. The prediction methods in this group focus on mining informative features consisting of GO terms [2-5], sorting signals [6,7], amino acid composition (AAC) [8-10], *k*-peptide encoding vector [7,11-14], physicochemical properties of amino acids [15-17], and fusing AAC and physicochemical properties [2,4,18,19].

b) Designing efficient classifiers. Most of the following prediction methods use effective classifiers based on support vector machine (SVM) [5,10-12,14,16,17,20] or the *k *nearest neighbour (*k*-NN) classifiers [2,4,5,13,19,21].

c) Integrating informative features with efficient classifier. Methods in this group include pSLIP [17], ProLoc [18], Euk-OET-PLoc [2] and Hum-PLoc [4]. The pSLIP system utilizes five top-rank features of physicochemical properties according to the prediction accuracy of SVM using a single feature [17]. The ProLoc system uses SVM with automatic selection from physicochemical properties to predict protein subnuclear localization [18]. The two ensemble classifiers Euk-OET-PLoc [2] and Hum-PLoc [4] fuse many basic individual classifiers operated by the engine of *k*-NN rules, where protein sequences are represented by hybridizing the GO annotation and amphiphilic pseudo amino acid (Pse-AA) composition.

Additionally, these two efficient GO-based systems Euk-OET-PLoc [2] and Hum-PLoc [4] predict subcellular localization of proteins using their known accession numbers. However, they cannot work for novel proteins without known accession numbers. The GO-AA method [5], which uses GO terms of homologies retrieved by BLAST to assess protein similarity, can deal with novel proteins without known accession numbers for subnuclear localization prediction. Besides, some SVM-based methods using only the features derived from input sequences, such as ProtLock with AAC [8], Ploc with AAC and acid pairs [10], and HSLPred with AAC and dipeptide composition [11], predict subcellular localization inaccurately [4]. Therefore, this study would develop an accurate SVM-based method for predicting subcellular localization of novel proteins by using input sequences with BLAST.

The Gene Ontology provided by the GO Consortium [1] has quickly grown in size and popularity. The newest version (UniProt 52.0 released in September 2007) of GO [22] contained 29,383 terms in the three branches, molecular function, biological process and cellular component. The terms and relationships among them are represented by a directed acyclic graph in which vertices represent the GO terms, and edges represent the relationships among these terms. Genes can be annotated with GO terms creating gene associations that can be used for whole genome analyses [23].

GO annotation has been successfully used in various sequence-based applications, which can be classified into two groups. 1) The first group uses the GO terms and their corresponding structure information of GO graph, such as grouping GO terms to improve the assessment of gene set enrichment [24]; using GO with probabilistic chain graphs for protein classification [25,26], and prediction of subnuclear localization [5]. 2) The second group uses GO terms only without structure information, such as predicting transcription factor DNA binding preference [27] and various predictions of subcellular and subnuclear localization [2,4,5]. In the second group, protein sequences are often represented as high dimensional vectors of *n *binary features, where *n *is the total number of terms in the complete annotation set (a component of 1 if the annotation is hit, and 0 otherwise) [28]. This representation is valuable in well-known vector space clustering algorithms such as *k*-NN [2,4,13,19,21] and fuzzy *k*-NN [13,29,30]. However, because *n *is often large, and each gene product is generally annotated by few GO terms, the vectors became long and sparse, making the clustering rather problematic [28].

This study proposes an efficient method, named GOmining, based on an intelligent genetic algorithm (IGA) [31,32] incorporating an SVM classifier to simultaneously identify a small number *m *out of a large number *n *of GO terms as input features, where *m *<<*n*. Some GO annotations corresponding to subcellular compartments are called essential GO terms for subcellular localization prediction, such as GO:0005634 (Nucleus), GO:0005737 (Cytoplasm) and GO:0005856 (Cytoskeleton), shown in Table 1. These essential GO terms are regarded as domain knowledge to be included in the feature set of *m *informative GO terms for subcellular localization prediction. A prediction method ProLoc-GO based on GOmining was implemented using the feature set of informative GO terms. This method performed well in predicting protein subcellular localization from input sequences only.

**Table 1:** Essential GO terms and their definitions

Compartment	Essential	Definition
	GO term	
Centriole	GO:0005814	A cellular organelle, found close to the nucleus in many eukaryotic cells, consisting of a small cylinder with microtubular walls, 300–500 nm long and 150–250 nm in diameter.
Cytoplasm	GO:0005737	All of the contents of a cell excluding the plasma membrane and nucleus, but including other subcellular structures.
Cytoskeleton	GO:0005856	Any of the various filamentous elements that form the internal framework of eukaryotic cells, and typically remain after treatment of the cells with mild detergent to remove membrane constituents and soluble components of the cytoplasm.
Endoplasmic reticulum	GO:0005783	The irregular network of unit membranes, visible only by electron microscopy, that occurs in the cytoplasm of many eukaryotic cells.
Extracellular	GO:0030198	A process that is carried out at the cellular level which results in the formation, arrangement of constituent parts, or disassembly of an extracellular matrix
Golgi apparatus	GO:0005794	A compound membranous cytoplasmic organelle of eukaryotic cells, consisting of flattened, ribosome-free vesicles arranged in a more or less regular stack. ...
Lysosome	GO:0005764	Any of a group of related cytoplasmic, membrane bound organelles that are found in most animal cells and that contain a variety of hydrolases, most of which have their maximal activities in the pH range 5–6. ...
Chloroplast	GO:0009507	Any of the small, heterogeneous, artifactual, vesicular particles, 50–150 nm in diameter, that are formed when some eukaryotic cells are homogenized and that sediment on centrifugation at 100000 g.
Microsome	GO:0005792	A semiautonomous, self replicating organelle that occurs in varying numbers, shapes, and sizes in the cytoplasm of virtually all eukaryotic cells. It is notably the site of tissue respiration.
Mitochondrion	GO:0005739	A membrane-bounded organelle of eukaryotic cells in which chromosomes are housed and replicated. ...
Nucleus	GO:0005634	A small, membrane-bounded organelle that uses dioxygen (O2) to oxidize organic molecules; contains some enzymes that produce and others that degrade hydrogen peroxide (H2O2).
Peroxisome	GO:0005777	The membrane surrounding a cell that separates the cell from its external environment. It consists of a phospholipid bilayer and associated proteins.
Plasma membrane	GO:0005886	A cellular organelle, found close to the nucleus in many eukaryotic cells, consisting of a small cylinder with microtubular walls, 300–500 nm long and 150–250 nm in diameter. It contains nine short, parallel, peripheral microtubular fibrils, each fibril consisting of one complete microtubule fused to two incomplete microtubules.
Cell wall	GO:0005618	The rigid or semi-rigid envelope lying outside the cell membrane of plant, fungal, and most prokaryotic cells, maintaining their shape and protecting them from osmotic lysis.
Cyanelle	GO:0009842	Plastid type found in Glaucophyta having unstacked thylakoid membranes bearing phycobilisomes; cyanelles are bound by a double membrane and a peptidoglycan layer.
Vacuole	GO:0005773	A closed structure, found only in eukaryotic cells, that is completely surrounded by unit membrane and contains liquid material.
Plastid	GO:0009536	Any member of a family of organelles found in the cytoplasm of plants and some protists, which are membrane-bounded and contain DNA.

## Results

### Data sets

Two existing data sets SCL12 [4] and SCL16 [2] obtained from UniProtKB/Swiss-Prot database [33] were used to evaluate the proposed method ProLoc-GO. The SCL12 and SCL16 have 2041 human proteins localized in 12 human subcellular compartments and 4150 eukaryotic proteins in 16 subcellular compartments, respectively. The two data sets were operated by a culling program [34] so that those sequences had < 25% sequence identity.

The proteins in SCL12 were screened strictly using the following rules: 1) only those sequences annotated with "human" in the ID (identification) field were collected; 2) sequences annotated with ambiguous or uncertain terms, such as "potential", "probable", "probably", "maybe", or "by similarity", were excluded; 3) sequences annotated by two or more locations were excluded, and 4) sequences with less than 50 amino acid residues were removed [4]. The data set SCL12 was divided into two parts, SCL12L and SCL12T, with 919 and 1122 proteins, respectively. The SCL12L set was used for training and the SCL12T was used for independent testing, as shown in Table 2[4].

**Table 2:** Data set SCL12. The data set SCL12 consists of SCL12L and SCL12T as the learning data set and testing data set, respectively. There are 12 essential GO terms corresponding to subcellular compartments. The number *t *of (*t*) in SCL12L represents the number of sequences which are correctly annotated by only one essential GO term.

Label	Compartment	Essential	Number of sequences	
		GO term	SCL12L	SCL12T
1	Centriole	GO:0005814	20 (1)	25
2	Cytoplasm	GO:0005737	155 (38)	377
3	Cytoskeleton	GO:0005856	12 (6)	14
4	Endoplasmic reticulum	GO:0005783	28 (19)	35
5	Extracellular	GO:0030198	140 (0)	301
6	Golgi apparatus	GO:0005794	33 (5)	42
7	Lysosome	GO:0005764	32 (27)	40
8	Microsome	GO:0005792	7 (0)	8
9	Mitochondrion	GO:0005739	125 (111)	228
10	Nucleus	GO:0005634	196 (179)	580
11	Peroxisome	GO:0005777	18 (16)	23
12	Plasma membrane	GO:0005886	153 (23)	368

Total			919 (425)	1122

The proteins of SCL16 were screened according to four criteria. The first criterion is to exclude sequences annotated with "prokaryotic", because this study focused only on eukaryotic proteins. The other three criteria were the same as criteria 2–4 for SCL12 above. Table 3 shows the numbers of proteins within each compartment, where the SCL16 consists of two parts, SCL16L for training and SCL16T for independent testing. The sequences in the training and test data sets were obtained from the web servers of Euk-OET-PLoc [2] and Hum-PLoc [4].

**Table 3:** Data set SCL16. The data set SCL16 consists of SCL16L and SCL16T as the learning data set and testing data set, respectively. There are 15 essential GO terms corresponding to eukaryotic subcellular compartments. Note that GO:0005814 is not appeared in the set of *n *= 2870 GO terms. The number *t *of (*t*) in SCL16L represents the number of sequences which are correctly annotated by only one essential GO term.

Label	Compartment	Essential	Number of sequences	
		GO term	SCL16L	SCL16T
1	Centriole	GO:0005814	17 (0)	4
2	Cytoplasm	GO:0005737	384 (92)	334
3	Cytoskeleton	GO:0005856	20 (7)	5
4	Endoplasmic reticulum	GO:0005783	91 (83)	22
5	Extracellular	GO:0030198	402 (1)	404
6	Golgi apparatus	GO:0005794	68 (8)	17
7	Lysosome	GO:0005764	37 (32)	9
8	Chloroplast	GO:0009507	207 (192)	51
9	Mitochondrion	GO:0005739	183 (173)	45
10	Nucleus	GO:0005634	474 (395)	695
11	Peroxisome	GO:0005777	52 (38)	12
12	Plasma membrane	GO:0005886	323 (29)	90
13	Cell wall	GO:0005618	20 (16)	5
14	Cyanelle	GO:0009842	78 (65)	19
15	Vacuole	GO:0005773	36 (30)	8
1 6	Plastid	GO:0009536	31 (1)	7

Total			2423 (1162)	1727

### GO annotation

This study applied the Gene Ontology Annotation (GOA) database [35], which includes GO annotations for non-redundant proteins from many species in the UniProtKB/Swiss-Prot database [33]. The GOA database was downloaded directly from [36] (UniProt 45.0 released in Jan. 2007). The accession numbers of proteins are required for querying the GOA database to obtain GO terms. BLAST [37,38] was used to obtain a homology with a known accession number to the protein for retrieving the GO terms. The corresponding accession numbers of all protein sequences in SCL12 and SCL16 were obtained by using BLAST with *h *= 1 and *e *= 10^-9^.

Table 4 shows the GO annotation results of all proteins in the training data sets SCL12L and SCL16L. For SCL12L, the size of the complete set of all GO terms that appeared was *n *= 1714 from the 919 human proteins. The smallest, largest and mean numbers of GO terms annotated for individual proteins were 0, 35 and 8.3, respectively. The percentage of training proteins whose homologies were not annotated by any GO term (that is, the number of GO terms annotated is zero) was 1.31%. For SCL16L, *n *= 2870 GO terms were obtained from 2423 eukaryotic proteins. The smallest, largest and mean numbers of GO terms annotated were 0, 50 and 7.7, respectively. The percentage of training proteins whose homologies were not annotated was 3.96%. The proteins annotated by GO are often represented as an *n*-dimensional binary feature vector, where the attribute value is 1 if the corresponding GO term is annotated, and 0 otherwise.

**Table 4:** Results of GO annotation for all sequences in SCL12L and SCL16

Data set	Total GO terms *n*	Number of GO terms			Number of sequences annotated by *g *essential GO terms		
		Smallest	Largest	Mean	*g *= 0	*g *= 1	*g *> 1
SCL12L	1714	0	35	8.3	404	453	62
SCL16L	2870	0	50	7.7	1025	1247	151

To know the prediction performance according to only the essential GO terms annotated, we calculated the numbers of sequences annotated by *g *essential GO terms. Table 4 shows that 453 out of 919 (49.3%) sequences are annotated by only one essential GO term (*g *= 1) for SCL12L, where 425 sequences are correctly annotated and 28 sequences are incorrectly annotated. The other 466 sequences annotated by zero (*g *= 0) or more than one (*g *> 1) essential GO term can not be effectively predicted. Table 2 lists the numbers of sequences which are correctly annotated by only one essential GO term for every compartment. The two GO terms, GO:0005634 (Nucleus) and GO:0005739 (Mitochondrion), made a great contribution to the prediction accuracy of 46.2% (= 425/919), which correctly annotate a large number of sequences, 179 and 111, respectively.

As for SCL16L, the number of sequences annotated by only one essential GO term is 1247 out of 2423 (51.5%). Table 3 lists the numbers of sequences which are correctly annotated by only one essential GO term for every compartment. Only 48.0% (= 1162/2423) of the sequences with known accession numbers can be correctly predicted by using only the annotation of essential GO terms. According to Table 3, the three essential GO terms, GO:0005634 (Nucleus, 395 out of 474), GO:0009507 (Chloroplast, 192 out of 207) and GO:0005739 (Mitochondrion, 173 out of 183), made a great contribution to prediction accuracy.

The analytic results reveal that it is not sufficient to use only essential GO terms for accurately predicting protein subcellular localization. However, the essential GO terms play an important role in designing GO-based prediction methods.

### Selected informative GO terms

Selecting a set of *m *informative GO terms out of *n *candidate GO terms is a combinatorial optimization problem C(*n*, *m*), which can be solved by using the intelligent genetic algorithm with an inheritance mechanism (IGA) [31,32]. IGA can efficiently search for the solution *S*_r+1 _to C(*n*, *r*+1) by inheriting a good solution *S*_r _to C(*n*, *r*). This study proposes an efficient algorithm based on IGA, called GOmining, to identify a small set of *m *informative GO terms including the essential GO terms as features to SVM. The GOmining algorithm incorporates LIBSVM [39] using series of binary classifiers. GOmining aims to maximize the training accuracy of prediction using 10-fold cross-validation (10-CV) when identifying the *m *informative GO terms.

**Figure 1:**
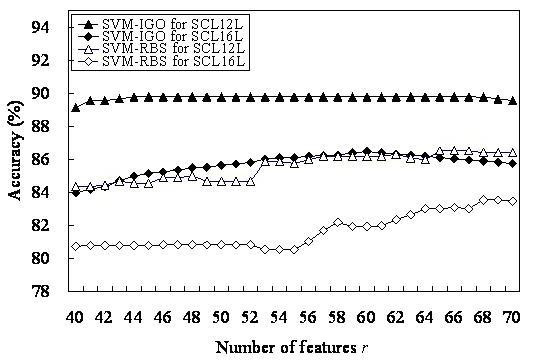
Training accuracies of SVM-IGO and SVM-RBS performed by using SVM with a number *r *of selected informative GO terms.

The SVM classifier based on the selected informative GO terms as features is called SVM-IGO. To evaluate a candidate set of *r *informative GO terms accompanied with the SVM parameters, the prediction accuracy of 10-CV serves as a fitness function of IGA. Figure 1 shows the results of SVM-IGO from *r *= 40, 41,..., 70. Table 5 lists the *m *= 44 informative GO terms for SCL12L obtained from the highest accuracy of 89.8% (*r *= 44), where the SVM parameters (*C*, *γ*) = (2^3^, 2^-4^). Table 6 lists the *m *= 60 informative GO terms for SCL16L, where the highest accuracy was 86.5%, and (*C*, *γ*) = (2^5^, 2^-3^).

**Table 5:** The *m *= 44 informative GO terms by applying GOmining to SCL12L. The GO terms in bold style are essential GO terms.

Rank by MED	GO term	Branch	MED	Rank by MED	GO term	Branch	MED
1	**GO:0005634**	C	390.1	23	GO:0007218	B	57.1
2	**GO:0005739**	C	350.3	24	GO:0042742	B	56.3
3	GO:0016021	C	297.6	25	GO:0005815	C	56.3
4	GO:0005576	C	136.6	26	GO:0005319	M	55.2
5	GO:0008285	B	72.8	27	GO:0020037	M	54.7
6	**GO:0005814**	C	70.2	28	**GO:0005792**	C	50.8
7	GO:0050909	B	69.3	29	**GO:0005856**	C	43.0
8	GO:0008633	B	69.3	30	GO:0005215	M	42.5
9	GO:0009396	B	67.4	31	**GO:0005764**	C	41.7
10	**GO:0030198**	B	66.9	32	GO:0016757	M	39.5
11	GO:0031227	C	66.7	33	**GO:0005737**	C	37.3
12	GO:0006888*	B	66.1	34	**GO:0005886**	C	36.0
13	GO:0005859	C	65.4	35	GO:0050896	B	33.6
14	**GO:0005794**	C	64.7	36	GO:0005813	C	30.6
15	GO:0009596	B	64.1	37	GO:0005578	C	28.8
16	GO:0006421	B	63.7	38	GO:0005615	C	28.4
17	GO:0006941	B	63.7	39	GO:0007165	B	22.7
18	GO:0005622	C	63.0	40	GO:0006886	B	20.1
19	GO:0004356	M	62.6	41	GO:0030662	C	19.9
20	GO:0008484	M	62.6	42	GO:0005216	M	9.7
21	GO:0017119	C	62.1	43	**GO:0005777**	C	3.8
22	GO:0006879	B	58.9	44	**GO:0005783**	C	1.2

**Table 6:** The *m *= 60 informative GO terms by applying GOmining to SCL16L. The GO terms in bold style are essential GO terms.

Rank by MED	GO term	Branch	MED	Rank by MED	GO term	Branch	MED
1	**GO:0005634**	C	331.7	31	GO:0005525	M	56.5
2	**GO:0009507**	C	244.2	32	GO:0005789	C	55.6
3	GO:0016020	C	148.1	33	GO:0004725	M	55.6
4	**GO:0005739**	C	147.8	34	GO:0008270	M	54.6
5	GO:0001844	B	70.7	35	GO:0015031	B	54.1
6	GO:0005212	M	70.5	36	GO:0005813	C	52.2
7	**GO:0005886**	C	68.5	37	GO:0005524	M	51.8
8	GO:0006094	B	67.9	38	GO:0051536	M	51.8
9	GO:0045261	C	67.5	39	GO:0016702	M	51.1
10	**GO:0009536**	C	66.6	40	GO:0005887	C	48.4
11	GO:0007010	B	65.8	41	GO:0005905	C	46.1
12	**GO:0030198**	B	65.4	42	**GO:0005794**	C	43.6
13	GO:0009626	B	65.4	43	GO:0005622	C	43.4
14	GO:0005047	M	64.6	44	GO:0005759	C	43.1
15	GO:0017134	M	64.1	45	**GO:0005856**	C	42.1
16	GO:0000287	M	63.3	46	GO:0016757	M	41.7
17	GO:0006888	B	61.7	47	**GO:0005618**	C	41.3
18	GO:0030234	M	61.7	48	**GO:0005783**	C	35.7
19	GO:0007323	B	61.0	49	**GO:0005773**	C	31.2
20	GO:0008083	M	60.8	50	**GO:0009842**	C	27.2
21	GO:0004521	M	60.3	51	GO:0006811	B	25.3
22	GO:0003723	M	60.2	52	GO:0006350	B	24.3
23	GO:0009514	C	59.9	53	GO:0016740	M	23.1
24	GO:0020015	C	59.4	54	**GO:0005737**	C	20.2
25	GO:0000922	C	59.0	55	GO:0005829	C	19.6
26	GO:0005681	C	58.9	56	GO:0016798	M	15.5
27	GO:0030149	B	57.9	57	GO:0007186	B	14.8
28	GO:0000917	B	56.9	58	GO:0019843	M	13.0
29	GO:0009405	B	56.8	59	**GO:0005764**	C	11.8
30	GO:0005615	C	56.5	60	**GO:0005777**	C	10.2

The orthogonal experimental design with orthogonal array and factor analysis used in IGA is an efficient method for simultaneously examining the individual effect of several factors on the evaluative function [40,41]. The factors are the parameters (GO terms) that manipulate the evaluation function, and a setting of a parameter is regarded as a level of the factor. In this study, the two levels of one factor are the inclusion and exclusion of the *i*th GO term in the feature selection using IGA. The factor analysis can quantify the effects of individual factors on the evaluation function, rank the most effective factors and determine the best level for each factor to optimize the evaluation function. The most effective factor has the largest main effect difference (MED). Tables 5 and 6 show that the essential GO term GO:0005634 (Nucleus) having the largest values of MED is the most effective feature of discrimination. The only essential GO term GO:0030198 (Extracellular matrix organization and biogenesis) belongs to biologic process branch and the other essential GO terms belong to cellular component branch. The abbreviations M, B and C represent the three branches molecular function, biological process, and cellular component, respectively.

### Evaluation of feature selection

SVM-IGO was implemented by using the *m *informative GO terms and the SVM classifier using (*C*, *γ*) = (2^3^, 2^-4^) and (*C*, *γ*) = (2^5^, 2^-3^) for SCL12L and SCL16L, respectively. To evaluate the effectiveness of SVM-IGO, four additional classifiers were implemented for comparison. Three classifiers SVM-GO, *k*-NN-GO and fuzzy *k*-NN-GO in order based on SVM, *k*-NN and fuzzy *k*-NN were implemented by using all the *n *GO terms as features without GO term selection. The classifier SVM-RBS used SVM with a subset of *n *GO terms selected by the rank-based selection (RBS) method [17,42]. The best values of parameters *C *and *γ *determined using a step-wise approach were employed to the SVM-based methods SVM-GO and SVM-RBS, where *γ *∈ {2^-7^, 2^-6^,..., 2^8^} and *C *∈ {2^-7^, 2^-6^,..., 2^8^}. The best values (*C*, *γ*) = (2^3^, 2^-4^) and (2^4^, 2^-6^) were applied to SVM-GO for SCL12L and SCL16L, respectively. As for SVM-RBS, (*C*, *γ*) = (2^1^, 2^-3^) and (2^2^, 2^-2^) were used for SCL12L and SCL16L, respectively. The Methods section describes SVM-RBS, *k*-NN-GO and fuzzy *k*-NN-GO in detail. Table 7 lists all prediction accuracies using 10-CV for both data sets SCL12L and SCL16L.

**Table 7:** Comparison of prediction accuracy (%) using 10-CV. Performance comparison uses prediction accuracy (%) of 10-CV.

Data set	*k*-NN-GO	Fuzzy *k*-NN-GO	SVM-GO	SVM-RBS	SVM-IGO
SCL12L	74.3	71.4	88.5	86.5	89.8
SCL16L	66.0	59.3	84.8	83.5	86.5

The highest accuracies of SVM-RBS are 86.5% and 83.5% using 65 and 68 selected GO terms for SCL12L and SCL16L, respectively, shown in Fig. 1. Table 7 shows that the three SVM-based classifiers (SVM-GO, SVM-RBS and SVM-IGO), with accuracies >80%, were better than the two *k*-NN based classifiers (*k*-NN-GO and fuzzy *k*-NN-GO), with accuracies <75%, for both data sets. SVM-IGO had the highest accuracies 89.8% and 86.5% for SCL12L and SCL16L, respectively. The GO term selection method based on GOmining was more effective than RBS and the method without selection of GO terms. Furthermore, SVM uses the selected GO terms as features, making it better than the *k*-NN classifier.

### Performance comparison

The proposed ProLoc-GO method predicts the subcellular localization of an input sequence using either SVM-IGO or SVM-GO, depending on its annotation on the informative GO terms (see Methods for detail). Tables 8, 9, 10, 11 list the results of ProLoc-GO using SCL12 and SCL16. Some existing AAC-based prediction methods, such as ProtLock [8], Least Euclidean distance [9], Ploc [10] and HSLPred [11], use only the query sequence as input data for their classifiers. Hum-PLoc [4] and Euk-OET-PLoc [2] use both the sequence and its accession number as input data. For comparison with these predictors, the method ProLoc-GO was performed using the two kinds of input data separately. The first test used only the sequence and used BLAST to obtain annotated GO terms. The second test used the known accession number of proteins directly. For the accuracy on both SCL12L and SCL16L, ProLoc-GO used leave-one-out cross-validation (LOOCV) for comparison with the other methods (see Methods section).

**Table 8:** Comparison of prediction accuracy (%) for SCL12. Prediction accuracies (%) for using leave-one-out cross-validation (LOOCV) on SCL12L and independent test on SCL12T are obtained from the paper [4]. The input data is sequence only (S) or sequence with accession number (AN).

Method	Input data	Features	LOOCV SCL12L	Independent test SCL12T
ProtLock [8]	S	AAC	29.7	26.4
Least Euclidean distance [9]	S	AAC	30.1	29.5
Ploc [10]	S	AAC and amino acid pairs	30.5	34.3
HSLPred [11]	S	AAC and dipeptide composition	30.7	33.3
ProLoc-GO	S	GO terms using BLAST	90.0	88.1

Hum-PLoc [4]	S with AN	Hybridization of GO terms and Pse-AA	81.1	85.0
ProLoc-GO	S with AN	GO terms (No BLAST)	91.1	90.6

**Table 9:** Comparison of prediction accuracy (%) for SCL16. Prediction accuracies (%) of using LOOCV on SCL16L and independent test on SCL16T are obtained from the paper [2]. The input data is sequence only (S) or sequence with accession number (AN).

Method	Input data	Features	LOOCV SCL16L	Independent test SCL16T
ProtLock [8]	S	AAC	28.7	25.3
Least Euclidean distance [9]	S	AAC	25.8	20.4
Ploc [10]	S	AAC and amino acid pairs	35.1	32.8
HSLPred [11]	S	AAC and dipeptide composition	33.1	34.5
ProLoc-GO	S	GO terms using BLAST	86.6	83.3

Euk-OET-PLoc [2]	S with AN	Hybridization of GO terms and Pse-AA	81.6	83.7
ProLoc-GO	S with AN	GO terms (No BLAST)	89.0	85.7

**Table 10:** Accuracies and MCC preformed on SCL12

Label	Compartment	SCL12L	SCL12T	
		Sequence	Sequence	Accession no.
1	Centriole	65.0 (0.803)	60.0 (0.670)	60.0 (0.774)
2	Cytoplasm	88.4 (0.784)	82.9 (0.734)	85.1 (0.790)
3	Cytoskeleton	16.7 (0.406)	0.0 (-0.002)	50.0 (0.314)
4	Endoplasmic reticulum	89.3 (0.804)	71.4 (0.501)	85.7 (0.603)
5	Extracellular	86.4 (0.871)	76.4 (0.802)	79.5 (0.830)
6	Golgi apparatus	48.5 (0.630)	33.3 (0.328)	44.4 (0.364)
7	Lysosome	96.9 (0.952)	87.5 (0.744)	87.5 (0.781)
8	Microsome	85.7 (0.800)	100.0 (0.446)	100.0 (0.446)
9	Mitochondrion	99.2 (0.986)	97.1 (0.978)	100.0 (0.995)
10	Nucleus	95.9 (0.939)	94.3 (0.930)	96.9 (0.952)
11	Peroxisome	94.4 (0.943)	100.0 (0.912)	100.0 (0.912)
12	Plasma membrane	96.1 (0.942)	90.7 (0.893)	91.6 (0.921)

Overall accuracy % (MCC)		90.0 (0.822)	88.1 (0.661)	90.6 (0.724)

**Table 11:** Accuracies and MCC preformed on SCL16

Label	Compartment	SCL16L	SCL16T	
		Sequence	Sequence	Accession no.
1	Centriole	61.1 (0.747)	66.7 (0.729)	50.0 (0.577)
2	Cytoplasm	72.9 (0.706)	74.6 (0.676)	72.8 (0.659)
3	Cytoskeleton	20.0 (0.363)	0.0 (-0.002)	0.0 (-0.003)
4	Endoplasmic reticulum	90.1 (0.841)	77.3 (0.707)	72.7 (0.678)
5	Extracellular	89.6 (0.778)	84.4 (0.738)	84.9 (0.796)
6	Golgi apparatus	63.2 (0.653)	41.2 (0.508)	41.2 (0.486)
7	Lysosome	97.3 (0.973)	100.0 (0.865)	100.0 (0.865)
8	Chloroplast	98.6 (0.989)	100.0 (0.990)	100.0 (0.990)
9	Mitochondrion	99.5 (0.963)	91.1 (0.879)	95.6 (0.877)
10	Nucleus	89.0 (0.913)	86.3 (0.865)	93.5 (0.924)
11	Peroxisome	98.1 (0.971)	100.0 (0.960)	100.0 (0.925)
12	Plasma membrane	86.4 (0.851)	88.9 (0.773)	82.2 (0.798)
13	Cell wall	75.0 (0.655)	60.0 (0.422)	80.0 (0.631)
14	Cyanelle	88.5 (0.939)	94.7 (0.973)	100.0 (1.000)
15	Vacuole	86.1 (0.847)	62.5 (0.790)	50.0 (0.706)
16	Plastid	51.6 (0.595)	42.9 (0.426)	42.9 (0.311)

Overall accuracy % (MCC)		86.6 (0.799)	83.3 (0.706)	85.7 (0.710)

The test accuracies for ProLoc-GO performed on the human protein data set SCL12L and SCL12T were 90.0% and 88.1%, respectively, where *m *= 44, and (*C*, *γ*) = (2^3^, 2^-4^) for SVM-IGO and SVM-GO. These results were much better than those (<35%) of the four sequence-based prediction methods [8-11] using only input sequences, shown in Table 8. Ploc [10] had the highest test accuracy of 34.3% among the four AAC-based methods.

The Matthews correlation coefficient (MCC) [5,12,18] values are usually employed while evaluating the performance on unbalanced datasets. In addition to the overall accuracy, the MCC values were also recorded due to the unbalance of numbers of proteins localized in the compartments, such as 196 of Nucleus vs. 7 of Microsome (Table 2). The MCC is defined as follows [5]:

(1) $$\begin{array}{cc} MCC_{c}=\frac{p_{c}s_{c}-u_{c}o_{c}}{\sqrt{\left(p_{c}+u_{c}\right)\left(p_{c}+o_{c}\right)\left(s_{c}+u_{c}\right)\left(s_{c}+o_{c}\right)}}, & c=1,2,\ldots ,N_{c}, \end{array}$$

where *p*_*c *_is the number of correctly predicted proteins of the location *c*, *s*_*c *_is the number of correctly predicted proteins not in the location *c*, *u*_*c *_is the number of under-predicted proteins, *o*_*c *_is the number of over-predicted proteins, and *N*_*c *_is the number of locations. The test MCC performances of ProLoc-GO were 0.822 and 0.661 for SCL12L and SCL12T, respectively. Table 10 presents the detailed results for individual compartments. The results of the five sequence-based methods reveal that the set of informative GO terms is more useful for protein subcellular localization than the AAC-based features.

### Performance of using known accession numbers

The accession number of each protein sequence in SCL12 and SCL16 was available in querying the GOA database. For comparison with the methods [2,4] based on the proteins with known accession numbers, ProLoc-GO using the known accession numbers of proteins as input data obtained test accuracies of 91.1% and 90.6% (MCC = 0.724) performed on SCL12L and SCL12T, respectively, where *m *= 56, (*C*, *γ*) = (2^2^, 2^-1^) for SVM-IGO and (*C*, *γ*) = (2^2^, 2^-4^) for SVM-GO. Hum-PLoc [4] using hybridization of GO terms and Pse-AA composition obtained training and test accuracies of 81.1% and 85.0% for SCL12L and SCL12T, respectively. The performance of ProLoc-GO using sequences or accession numbers as the input data was better than that of Hum-PLoc [4] using the ensemble classifiers with features of both sequence and accession number.

Tables 9 and 11 show the performance results of ProLoc-GO and Euk-OET-PLoc [2] using SCL16. ProLoc-GO using input sequences yielded test accuracies 86.6% (MCC = 0.799) and 83.3% (MCC = 0.706) for SCL16L and SCL16T, respectively, where *m *= 60, (*C*, *γ*) = (2^5^, 2^-3^) for SVM-IGO, and (*C*, *γ*) = (2^4^, 2^-6^) for SVM-GO. ProLoc-GO is significantly better than all the AAC-based methods with test accuracies smaller than 35%. ProLoc-GO yields the test accuracies 89.0% and 85.7% (MCC = 0.710) for SCL16L and SCL16T, respectively, using the known accession numbers of proteins, where *m *= 60, (*C*, *γ*) = (2^2^, 2^-3^) for SVM-IGO and (*C*, *γ*) = (2^3^, 2^-5^) for SVM-GO. Euk-OET-PLoc [2] using the ensemble classifiers with features of both sequence and accession number obtains training and test accuracies of 81.6% and 83.7%, respectively. ProLoc-GO performed better than Euk-OET-PLoc on SCL16 using either sequences or accession numbers as the input data [2].

### Analysis of informative GO terms

The GOmining method identifies a feature set of *m *effective GO terms, called informative GO terms, to design an accurate SVM-based prediction method. Table 12 shows the distribution of the *m *informative GO terms in the GO graph. For SCL12L with *m *= 44, GOmining selected 12 essential GO terms and 32 instructive GO terms. The 32 instructive GO terms consist of 7 GO terms from the molecular function branch, 14 terms from the biological process branch, and 11 terms from the cellular component branch, denoted as 7(M), 14(B) and 11(C), respectively. Analytical results reveal that all the three branches contain instructive GO terms.

**Table 12:** Distribution of the *m *informative GO terms. Most instructive GO terms (80%) are not offspring of the essential GO terms that the ratios are 26/32 and 36/45 for SCL12L and SCL16L, respectively.

	SCL12L (*m *= 44)	SCL16L (*m *= 60)
Essential GO terms	12: 1 (B), 11 (C)	15: 1(B), 14(C)
Instructive GO terms:	32:	45:
(a) offspring of some essential GO term	4 (C)	9 (C)
(b) between two essential GO terms	2 (C)	0
(c) not offspring of any essential GO term	7(M), 14(B), 5 (C)	18(M), 13(B), 5(C)

Due to the high correlation among GO terms in the GO graph, the feature selection of SVM should consider simultaneously a set of informative GO terms, rather than individual GO terms. Since the essential GO terms are always included, GOmining benefits from a confined search space of candidate instructive GO terms. Considering the position relationships between instructive and essential GO terms in the GO graph, instructive GO terms belonged to one of the three classes: (a) offspring but not ancestor of some essential GO term; (b) between two essential GO terms, and (c) not offspring of any essential GO term. Of the 32 instructive GO terms, 4, 2 and 26 GO terms belonged to the classes (a), (b) and (c), respectively. The 26 GO terms consist of 7(M), 14(B) and 5(C). The GO terms near the root of the GO graphs are considered to be more generic while terms near the leaves are more specific [23]. Of the instructive GO terms, 81.2% (26/32) were not offspring of any essential GO term. These analytical results reveal that the essential GO terms are informative enough in predicting subcellular localization, and are effective in confining the space of searching instructive GO terms. The other six instructive GO terms from the cellular component branch have more specific functions than the essential GO terms in discrimination of the subcellular localization.

**Figure 2:**
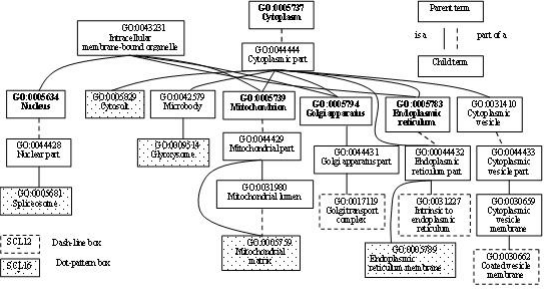
**Some of the selected GO terms which are offspring of essential GO terms**. For SCL12L, there are three terms shown: GO:0031227, GO:0030662 and GO:0017119. For SCL16L, five GO terms are shown: GO:0009514, GO:0005681, GO:0005789, GO:0005759 and GO:0005829.

**Figure 3:**
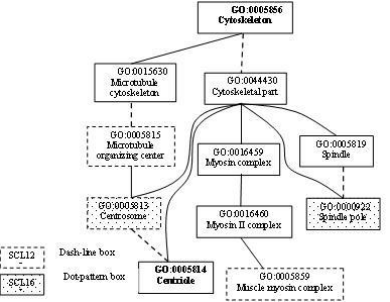
**Some of the selected GO terms which are between two essential GO terms**. For SCL12L, the two instructive GO terms GO:0005815 and GO:0005813 are between the essential GO terms GO:0005856 and GO:0005814. For SCL16L, GO:0005813 and GO:0000922 are offspring of the essential GO term GO:0005856, belonging to the class (a). GO:0005814 is not an essential GO term for SCL16L.

**Figure 4:**
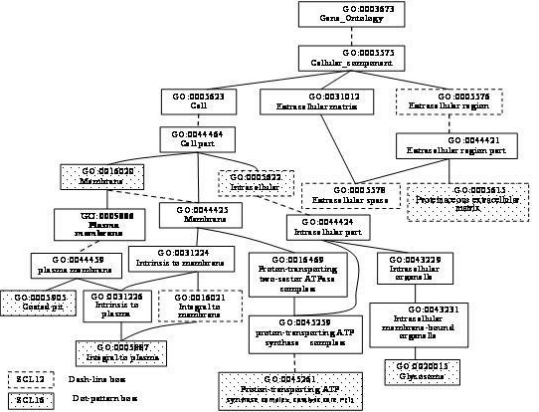
**Some of the selected GO terms are NOT offspring of any essential GO terms**. For SCL12L, five instructive GO terms are shown belonging to cellular component branch: GO:0016021, GO:0005576, GO:0005622, GO:0005578 and GO:0005615, which are not offspring of essential GO terms. For SCL16L, five GO terms belonging to the class (c) are shown: GO:0005622, GO:0005615, GO:0020015, GO:0016020 and GO:0045261. GO:0005905 and GO:0005887 belong to the class (a).

Figures 2, 3, 4 illustrate some of the instructive GO terms belonging to the three classes. Three instructive GO terms were found to belong to class (a), namely SCL12L: GO:0031227 (Intrinsic to endoplasmic reticulum membrane, rank 11), GO:30662 (Coated vesicle membrane, rank 41) and GO:0017119 (Golgi transport complex, rank 21), according to Fig. 2. The two terms belonging to class (b), namely GO:0005815 (Microtubule organizing center, rank 25) and GO:0005813 (Centrosome, rank 36), were found between the essential GO terms GO:0005856 (Cytoskeleton) and GO:0005814 (Centriole), as shown in Fig. 3. According to Fig. 4, five instructive GO terms belonging to the class (c) were not offspring of essential GO terms, GO:0016021 (Integral to membrane, rank 3), GO:0005576 (Extracellular region, rank 4), GO:0005622 (intracellular, rank 18), GO:0005578 (Proteinaceous extracellular matrix, rank 37) and GO:0005615 (Extracellular space, rank 38).

The *m *= 60 informative GO terms for SCL16L comprises 15 essential GO terms and 45 instructive GO terms. The 45 instructive GO terms consisted of 18(M), 13(B) and 14(C). The numbers of instructive GO terms coming from each branch were not significantly different. However, the numbers of instructive GO terms belonging to the three classes (a), (b) and (c) are 9, 0 and 36, respectively, which are very different. 80% (36/45) of the instructive GO terms were not offspring of any essential GO term. The 9 instructive GO terms belonging to the class (a) had 5, 2 and 2 terms, respectively, as shown in Figs. 2, 3 and 4. Class (c) has five GO terms with a dot-pattern box: GO:0005622 (intracellular), GO:0005615 (Extracellular space), GO:0020015 (Glycosome), GO:0016020 (Membrane) and GO:0045261 (Proton-transporting ATP synthase complex, catalytic core F(1)), as revealed by Fig. 4.

The statistical results of instructive GO terms distributed in the three classes for both SCL12L and SCL16L reveal that the inclusion of essential GO terms can be regarded as using domain knowledge for GOmining to mine a feature set of informative GO terms. The heuristic approach (using domain knowledge) of GOmining is efficient when the GO database grows fast. Therefore, GOmining can be easily applied to other applications of sequence-based predictions using SVM with the features of informative GO terms.

## Discussion

The GO database has grown in size recently, increasing the effectiveness of GO-based features. Meanwhile, the percentage of proteins with subcellular locations annotated in the GO database increased from 44.9% [2] to 65.5% [3] fast. It is indicated that there is a linkage in the GO annotation process between molecular function annotation and subcellular localization annotation [43]. Therefore, the GO-based prediction method for protein subcellular localization is increasingly efficient. Because the accession number of proteins is necessary for retrieving GO terms from GO databases, existing efficient GO-based systems Euk-OET-PLoc [2] and Hum-PLoc [4] directly utilize the accession numbers of proteins and a large number *n *of GO terms annotated in a complete set where *n *= 9918 for SCL12L [4] and *n *= 9567 for SCL16L [2].

To predict subcellular localizations for novel proteins, ProLoc-GO uses a good homology, rather than the query protein itself, to retrieve annotated GO terms using BLAST. To use GO term features effectively, ProLoc-GO uses only a homology with annotated GO terms to reduce *n*. Thus, *n *= 1714 for SCL12L and *n *= 2870 for SCL16L. Furthermore, a small set of *m *informative GO terms is selected simultaneously by GOmining. GOmining can consider internal relevant-feature correlation, instead of individual features by using an efficient global optimization method. The distribution analysis of informative GO terms in the GO graph is consistent with the properties of GO annotation. Additionally, ProLoc-GO using input sequences is slightly worse than using the accession numbers of proteins, with accuracies of 88.1% vs. 90.6% for SCL12T, and 83.3% vs. 85.7% for SCL16T, as shown in Tables 10 and 11.

## Conclusion

Computational prediction methods from primary protein sequences are fairly economical in terms of identifying large-scale eukaryotic proteins with unknown functions. The GO annotation, which describes the function of genes and gene products across species, has been used to improve the prediction of protein subcellular localization. The accession numbers of proteins are necessary to query the GOA database to obtain GO terms. Since novel proteins have no known accession numbers, BLAST was used to obtain homologies with known accession numbers to the proteins for the retrieval of GO terms.

GO annotation has grown in size and popularity. However, few studies have explored informative GO terms from the over 20,000 annotations available at present for sequence-based prediction problems. This study proposes a genetic algorithm based method, GOmining, which combines SVM to simultaneously identify a small number *m *out of the *n *GO terms as features to SVM, where *m *<<*n*. The *m *GO terms include the essential GO terms annotating subcellular compartments such as GO:0005634 (Nucleus), GO:0005737 (Cytoplasm) and GO:0005856 (Cytoskeleton). ProLoc-GO was evaluated using SVM with the GO-based features from two kinds of input data, sequence and known accession numbers of proteins.

ProLoc-GO yields test accuracies of 88.1% and 83.3% from SCL12 and SCL16, respectively, when using only input sequences. These results are significantly superior to those of the other SVM-based methods, which have accuracies <35% using AAC with acid pairs, and using AAC with dipedtide composition. ProLoc-GO using known accession numbers of proteins has accuracies 90.6% and 85.7% for SCL12 and SCL16, which is also slightly better than Hum-PLoc and Euk-OET-PLoc, which have 85.0% and 83.7%, respectively.

Analysis of *m *informative GO terms in the GO graph reveals that GOmining can consider internal relevant-feature correlation, rather than individual features, by using an efficient global optimization method. GOmining can serve as an efficient tool for mining informative GO terms for various sequence-based predictions of proteins, especially when the GO database grows fast. The prediction system using ProLoc-GO with protein sequence as input data for protein subcellular localization has been implemented (see Availability).

## Methods

### Proposed GOmining algorithm

An efficient genetic-algorithm-based method, called GOmining, is proposed for selecting informative GO terms. GOmining uses an intelligent genetic algorithm with an inheritable mechanism (IGA) [31,32], combined with an SVM classifier, to simultaneously identify a small number *m *out of a large number *n *of GO terms as input features, where *m *<<*n*. The exploration of the *m *informative GO terms from *n *candidate GO terms is a combinatorial optimization problem C(*n*, *m*) with a huge search space of size C(*n*, *m*) = *n*!/(*m*!(*n*-*m*)!)). An IGA based on orthogonal experimental design using a divide-and-conquer strategy and systematic reasoning method can efficiently solve this large combinatorial optimization problem.

The leave-one-out cross-validation (LOOCV) is considered to be the most rigorous and objective test. Although bias-free, this test is very computationally demanding and is often impractical for large data sets. The *N*-fold cross-validation not only provides a bias-free estimation of the accuracy at a much reduced computational cost, but is also considered as an acceptable test for evaluating prediction performance of an algorithm [44]. Therefore, GOmining uses the prediction accuracy of 10-CV as the fitness function to perform IGA on the entire training sets of proteins under considering the computation cost.

The input of the algorithm GOmining is composed of 1) a training set of protein sequences categorized into a number of compartments (classes), and 2) the essential GO terms corresponding to the compartments. The output comprises a set of *m *informative GO terms and the associated parameter settings of an SVM classifier. Since the novel sequences without known accession numbers use BLAST to obtain annotated GO terms, all training sequences use the same BLAST to obtain GO terms for consistence.

Step 1: (preparation of SVM) The multi-classification problem is solved by using a series of binary classifiers of LIBSVM [39]. In this study, the kernel parameter *γ *and cost parameter *C *are tuned where *γ *∈ {2^-7^, 2^-6^,..., 2^8^} and *C *∈ {2^-7^, 2^-6^,..., 2^8^}.

Step 2: (sequence representation) Obtain annotated GO terms from the GOA database for all training proteins using BLAST with *h *= 1 and *e *= 10^-9^. Let *n *be the total number of GO terms that appear among all proteins in the training data set. For example, *n *= 1714 and *n *= 2870 were derived for SCL12L and SCL16L, respectively. The protein is represented as an *n*-dimensional binary feature vector.

Step 3: (inclusion of essential GO terms) Identify *d *essential GO terms out of *n *GO terms and number them from 1 to *d*. For example, *d *= 12 and *d *= 15 were found from SCL12L and SCL16L, respectively.

**Figure 5:**
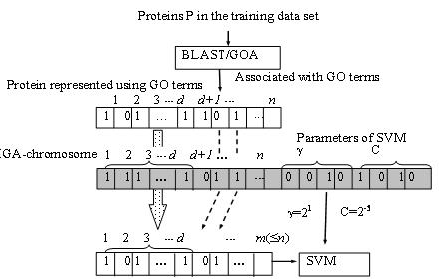
Sequence representation and IGA-chromosome encoding method.

Step 4: (chromosome encoding) The IGA-chromosome comprises *n *binary IGA-genes *f*_*i *_for selecting informative GO terms and two 4-bit IGA-genes for encoding *γ *and *C*, where *f*_*i *_= 1, *i *= 1,..., *d*. The *i*th GO term is included in the feature set of the SVM classifier if *f*_*i *_= 1; otherwise, the *i*th GO term is excluded (*f*_*i *_= 0). Figure 5 shows the sequence representation and IGA-chromosome encoding method.

Step 5: (initial solution) Perform IGA to select *r*_start _out of *n *GO terms, i.e., the solution to C(*n*, *r*_start_), where the *d *GO terms are always selected. Table 13 shows the parameter settings of IGA, such as crossover probability *p*_c _= 0.8. The procedure of IGA is described in detail in the work [18].

**Table 13:** The used control parameters of IGA

Parameter	Value
Population size *N*_pop_	50
Selection probability *p*_s_	0.2
Crossover probability *p*_c_	0.8
Mutation probability *p*_m_	0.05
Factor number of orthogonal arrays	7
Maximum generations *G*_max_	60

Step 6: (inheritance mechanism) The inheritance mechanism of IGA can efficiently search for the solution to C(*n*, *r*+1) by inheriting a good solution *S*_*r *_to C(*n*, *r*). Obtain all solutions *S*_*r *_from *r *= *r*_start_+1,..., *r*_end _one by one using IGA [31,32]. For example, *r*_start _= 40 and *r*_end _= 70 according to former experience.

Step 7: (decoding chromosome) Let *S*_*m *_be the most accurate solution with *m *selected GO terms among all solutions *S*_*r*_. Obtain the *m *informative GO terms and parameter values of *γ *and *C*.

Step 8: (robust performance) Perform Steps 5–7 for *N *independent runs to obtain the best one of *N *solutions *S*_*m *_and the associated parameter settings of the SVM parameters. The best solution considers both high prediction accuracy and high mean frequency of the *m *selected GO terms appeared in the *N *runs. In this study, *N *= 30.

### ProLoc-GO

**Figure 6:**
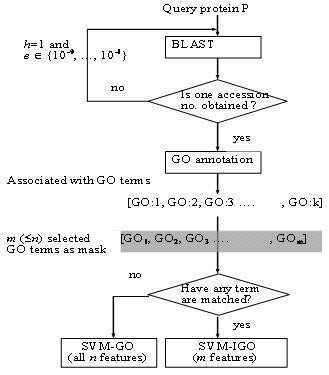
Prediction flowchart of ProLoc-GO using both classifiers SVM-IGO and SVM-GO.

As shown in Fig. 6, each query protein is first BLASTed with *h *= 1 and *e *= 10^-9 ^against the Swiss-Prot database to obtain a homology with a known accession number. If no such homology exists, then adjust the threshold value *e *of BLAST until the desired homology is obtained, where *h *= 1 and *e *∈ {10^-9^, 10^-8^,..., 10^-1^}. The accession number of the homology of each protein sequence in SCL12 and SCL16 was obtained by using BLAST with *h *= 1 and *e *= 10^-9^. This accession number is used as input to the GOA database for retrieving the corresponding *k *(>1) GO terms: GO:1, GO:2,... GO:k. If none of the *k *GO terms belongs to the set of *m *informative GO terms, then the sequence is represented using an *n*-dimensional binary vector and is predicted by the SVM-GO classifier. Otherwise, the sequence is represented as an *m*-dimensional binary vector and is predicted by the SVM-IGO classifier. Notably, the SVM-GO classifier predicts only a very small percentage of input sequences. ProLoc-GO is derived from the two major classifiers SVM-GO and SVM-IGO for subcellular localization prediction.

### Fuzzy k-NN

The protein is represented as an *n*-dimensional binary vector and the generalized distance between two proteins **P **and **P**_*i *_[2] is denoted as :

(2) *D*(**P**, **P**_*i*_) = 1 - **P**·**P**_*i*_/||**P**|| ||**P**_*i*_||,

where **P**·**P**_*i *_is the dot product of vectors **P **and **P**_*i*_, and ||**P**|| and ||**P**_*i*_|| are their moduli.

This study determined the best value of *k *by using a step-wise approach where *k *∈ {1, 2,..., 10}.

The fuzzy *k*-NN classifier [13,29,30] is a variation of *k*-NN, which assign fuzzy membership values *r*_c_(**P**) of a query sequence **P **to each class *c *as follows:

(3) $$\begin{array}{cc} r_{c}(P)=\frac{\sum_{j=1}^{k}r_{c}\left(P^{j}\right){\left|P-P^{j}\right|}^{-2/\left(w-1\right)}}{\sum_{j=1}^{k}{\left|P-P^{j}\right|}^{-2\left(w-1\right)}}, & c=1,2,...,N_{C}, \end{array}$$

where the distance is calculated by according to (1). In this study, the best values of parameters (*k*, *w*) are tuned iteratively from *k *∈ {1, 2,..., 10} and *w *∈ {1.05, 1.10,..., 1.95} for the fuzzy *k*-NN classifier.

### SVM-RBS

To evaluate the proposed IGA-based feature selection method GOmining, this study implements a classifier SVM-RBS by using SVM with a subset of the *n *GO terms by the rank-based selection (RBS) method [17,42]. One previous work on ProLoc [18] showed that this univariate method RBS is inferior to the multivariate feature selection by IGA for selecting physicochemical properties. First, each of all *n *GO terms (for example, *n *= 1714 for SCL12L) is ranked according to the accuracy of SVM with the evaluated single feature, where the best values of parameters (*C*, *γ*) were determined using a step-wise approach where *γ *∈ {2^-7^, 2^-6^,..., 2^8^} and *C *∈ {2^-7^, 2^-6^,..., 2^8^}. The top-ranking 70 features *a*_i_, *i *= 1,..., 70 are then picked, and the top-ranking 40 features with *r *= 40 are used as an initial feature set {*b*_1_,..., *b*_40_}. Consequently, the feature set with size *r*+1 is incrementally established by adding the best feature *b*_*r*+1 _(having the highest accuracy of SVM using 10-CV) from the remaining 70-*r *features into the current feature set.

## Authors' contributions

WLH designed the system, implemented programs, participated in manuscript preparation and carried out the detail study. CWT designed the system and implemented programs. SWH, SFH and SYH conceived the idea of this work. Additionally, SYH supervised the whole project and participated in manuscript preparation. All authors have read and approved the final manuscript.

## Availability

The prediction system using ProLoc-GO with input sequences of query proteins for protein subcellular localization has been implemented at .
